# Estimation of Fine and Oversize Particle Ratio in a Heterogeneous Compound with Acoustic Emissions

**DOI:** 10.3390/s18030851

**Published:** 2018-03-13

**Authors:** Ejay Nsugbe, Cristobal Ruiz-Carcel, Andrew Starr, Ian Jennions

**Affiliations:** School of Aerospace Transport and Manufacturing Building 50, College Rd, Cranfield University, Cranfield MK43 0AL, UK; c.ruizcarcel@cranfield.ac.uk (C.R.-C.); a.starr@cranfield.ac.uk (A.S.); i.jennions@cranfield.ac.uk (I.J.)

**Keywords:** particle size, process monitoring, time domain, online, acoustic emissions, heterogeneous compound, real time

## Abstract

The final phase of powder production typically involves a mixing process where all of the particles are combined and agglomerated with a binder to form a single compound. The traditional means of inspecting the physical properties of the final product involves an inspection of the particle sizes using an offline sieving and weighing process. The main downside of this technique, in addition to being an offline-only measurement procedure, is its inability to characterise large agglomerates of powders due to sieve blockage. This work assesses the feasibility of a real-time monitoring approach using a benchtop test rig and a prototype acoustic-based measurement approach to provide information that can be correlated to product quality and provide the opportunity for future process optimisation. Acoustic emission (AE) was chosen as the sensing method due to its low cost, simple setup process, and ease of implementation. The performance of the proposed method was assessed in a series of experiments where the offline quality check results were compared to the AE-based real-time estimations using data acquired from a benchtop powder free flow rig. A designed time domain based signal processing method was used to extract particle size information from the acquired AE signal and the results show that this technique is capable of estimating the required ratio in the washing powder compound with an average absolute error of 6%.

## 1. Introduction

The expression “particle size” typically refers to a physical feature of particles whose diameters range from nanometres to millimetres [1]. For powder samples that contain particles of different sizes, the various sizes present in the mixture and their associated amounts are usually presented in the form of a particle size distribution (PSD). In powder processing plants, the PSD is regarded as a critical factor due to its influence on the final bulk and flow properties of the particles [2,3,4]. This has led to a high level of interest in particle size measurement technologies, which has been covered in the review study conducted by Nsugbe et al. [5]. The choice of a particle size measurement sensor is partly dependent on the accuracy of the sensor and is highly dependent on the process environment factors, such as overall process hostility, sampling interval and presence of low-frequency acoustic activity [5]. In previous investigations, technologies such as electrostatic sensing, imaging, sieving and weighing, near-infrared spectroscopy (NIR), acoustic emission (AE), focused beam reflectance measurement (FBRM), microwave and vibration have been used [5]. For this investigation, acoustic emission (AE) was chosen as the sensing method to investigate the problem due to the benefit of being a non-invasive sensor that is insensitive to low-frequency acoustic activity and that it possesses primary sensitivity to frequencies at which particle events occur [2,5].

Notable research involving particle size monitoring with AE began with Leach et al. [6,7,8] who conducted experiments using a condenser microphone and a set of particles in a rotating drum. The results from their work showed a correlation between particles of different sizes and the resulting AE amplitude [6,7,8]. Buttle et al. [9] were able to carry out further experiments that validated Leach’s hypothesis and formed a mathematical expression for this relationship [9]. AE has also been used online to monitor particle sizes; Bastari et al. [3] used neural network-based systems to estimate particle sizes in a coal milling process, while Hu et al. [10] and Ren et al. [4] used a more hybrid signal processing approach to particle size measurement in their respective processes [3,4,10]. Hu et al. [10] used a time domain based threshold approach to estimate particle sizes in a pneumatically conveyed setup, while Ren et al. [4] used the AE energy of the particles by way of the wavelet transform to estimate the particle sizes in a fluidised bed [4,10].

Washing powder is a heterogeneous compound that comprises a mixture of 12 different particle types, each with unique physical properties, combined to form a single compound. Authors who have designed online particle size monitoring platforms, despite dealing with mixtures, have not investigated the characterisation of the size distribution of a compound consisting of different particle types. Ren et al. [4] considered a seven-particle mixture comprising differently sized polyethylene particles with different densities; however, as the physical properties of the particles were the same, other AE amplitude-dependent features such as Young’s modulus and Poisson’s ratio would be the same [4]. Bastari et al. [3]—who dealt with the sizing of a mixture online—used coal particles of different sizes for their experiments, while Hu et al. [10] dealt with a mixture of glass bead particles [3]. Thus, it can be inferred from the literature uncovered that the experiments in this report represent the first investigation into estimating the size distribution of a heterogeneous compound comprising different particles with various physical properties, and produce a signal from the simultaneous impact of particles hitting a target medium [11,12]. The focus of this paper involves the investigation of the feasibility of using an AE-based sensing approach to characterise particle sizes of a heterogeneous mixture using a designed benchtop experimental rig.

The work done in this study sees for the first time the use of the signal processing method to analyse AE data from a heterogeneous compound of this kind. The signal processing method is based on a time domain based hybrid signal processing method, which, due to its architecture, is believed to be capable of providing quicker online computation than its predecessors in the literature, such as the wavelet approach and the neural network method [3,4,5].

This paper is structured as follows: Section 2 discusses the offline check procedure carried out by the partner company Procter and Gamble, the rationale behind selecting AE, the benchtop experimental rig and the experimental method. Section 3 explains the theoretical model behind the sizing of particles with AE and details the design of the signal processing method to be used in this paper. Section 4 covers the experimental results and discussion and Section 5 summarises and concludes based on findings from the work detailed.

## 2. Materials and Method

### 2.1. Offline Check Procedure

Regarded as the oldest method of particle size measurement, the sieving method involves the separation of particles based on their sizes alone [13]. Typically, a sieving process consists of putting a measured mass of powder on a sieve with a known aperture size and providing the sieve with a force that enables the particles to pass through the sieve apertures [13]. If a broad characterisation of the sizes of the particles in the mixture is required, then sieves can be stacked on each other and is then regarded as a layer sieve [13]. The amount in each layer is weighed at the end of the process and calculated as a percentage of the total mass of the particles put in the sieve and used to form a PSD.

Procter and Gamble’s quality check procedure involves two phases, both of which take place offline. The first phase is the chemical test that is used to evaluate the chemical properties of the powders and involves a wet chemistry test, which is out of the scope of this paper as this report focuses on the physical checks carried out on the powders. The second phase involves the characterisation of the particle sizes by means of sieving. For Procter and Gamble, during the manufacture of the washing powder compound, the mixing and agglomeration process is the final stage before packaging commences. This mixing takes place in a batch mixer and at regular intervals the samples are extracted from a localised area of the finished mixture and the size distribution is characterised using sieve analysis. The sieve analysis involves the measurement of the ratio of fine particles to oversize particles in the sample mixture. The fine particles refer to particles in the range of 53–500 microns while particles in the range of 501–1500 microns are referred to as oversize particles. The obtained ratio from the sieve analysis procedure is used to form a PSD plot that, in addition to the wet chemistry test, is used as a reference point by the plant workers and a feedback indicator as to the quality of the powder batches being produced.

Although the sieving process has a set of distinct advantages, such as not requiring sample preparation and not requiring expert knowledge, there are also some distinct disadvantages of this measurement method [1,2]. Only a certain size of sample can be analysed by sieving and, therefore, the results offered by means of such analysis only reflect a localised area of the mixture [1]. Powder agglomerates with a considerable moisture content in certain cases cannot be analysed by sieving due to sieve blockage, and certain types of powders with strong electrostatic and van der Waals interactions between them are prone to erroneous sieve characterisation due to cohesive forces between the particles [2]. Another disadvantage is that being an offline process, sieve analysis does not allow the plant operators to have a closed-loop approach to their powder mixing process. The factors mentioned represent the challenges of the sieving method for characterising particle sizes and have necessitated the need from Procter and Gamble for real-time process monitoring sensors.

#### Sensor Selection

AE in the ultrasonic region was chosen as the sensing method to support the experiments in this study. This method was chosen due to its non-invasive nature, which means that the plant operators would not be required to carry out equipment modification in order to implement AE sensing. With the sensing frequency being in the ultrasonic region, it would provide primary sensitivity to particle events and insensitivity to the low-frequency acoustic activity produced by the process equipment. Another advantage is the low hardware cost associated with the implementation of the sensing method [2,5]. AE signals are acquired as the mixing process takes place and the powders make contact with the internal walls of the mixer—hence, sample extraction is not necessary. In addition, acquired AE signals are representative of the process as a whole and not a localised area; thus, the possibility of obtaining an erroneous PSD estimate due to powder segregation is greatly reduced when compared to sieving [2,5].

In contrast to the traditional sieving method, the availability of online AE sensing would help in creating an online closed-loop process, which in the future may allow for optimisation of the mixing process based on real-time feedback provided by the sensor.

The challenge with AE sensing remains the task of formulating signal processing algorithms capable of extracting the necessary information regarding particle size from a continuous AE signal containing particle events [14].

### 2.2. Materials and Setup

#### 2.2.1. Experimental Setup

The experimental rig used is based on the free fall of a known mass of powder on a target medium. This setup was chosen to simulate a controlled environment similar to the batch mixing process where particles are conveyed at a given rate and impact on the inside of the walls of the mixer.

**Figure 1:**
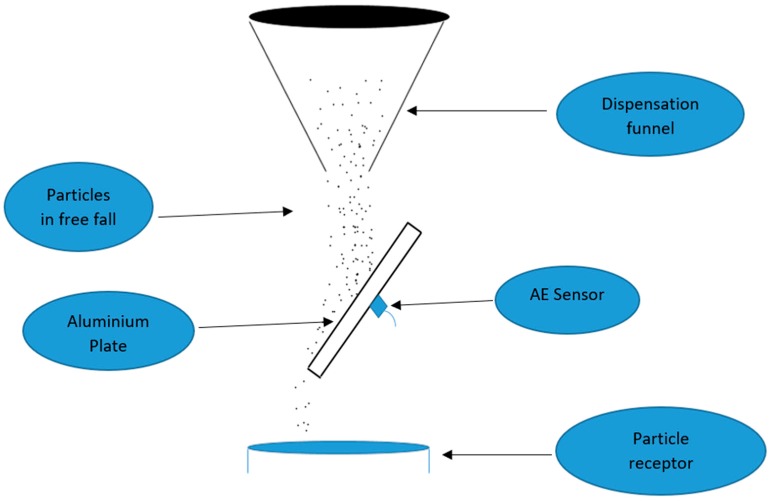
Diagram illustrating a side view of the experimental setup [12].

The rig was designed using a careful design of experiment (DOE) sequence, which helped ensure that the key attributes possessed by the industrial mixer were retained even though the benchtop rig had been simplified to help reduce operating variables. A diagram of the benchtop experimental rig can be seen in Figure 1.

The rig consisted of an aluminium plate measuring 11 × 18 cm^2^ with a density of 2.7 g/cm^3^, Poisson’s ratio of 0.35, Young’s modulus of 69 GPa and a uniform thickness of 0.7 mm, which served as a wave propagation medium. The sensor was attached to the back of the plate using a beeswax adhesive and the aluminium plate was inclined to prevent powder build up in and around the impact area. The particle dispensation funnel was hung at a height of 12 cm above the plate to enable particles to obtain a terminal velocity prior to their impact on the target plate [15,16]. The selected funnel, which produced a dispensation rate of 19.3 g/s, was chosen after a selection process where it was observed that some other funnels produced a bimodal flow rate in a random manner. The candidate funnel produced a unitary flow rate and this was essential to ensure that variations in the acquired signal emanated from a change in particle size and not from a variation in flow rate.

The sensor used was a structure-borne sensor with a bandwidth of 100 K–1 MHz and a sampling rate of 1 Ms/s was used during the acquisition. This sampling rate was chosen in order to ensure that the sensors acquired data points in the region where particle events are thought to emanate and prevent any form of aliasing from occurring where details from the full AE event would not be present in the acquired signal. During preliminary tests using particles of different materials and sizes, it was observed that the vast majority of the AE activity detected by the sensor was in the range of 100–500 kHz. The Fast Fourier Transform (FFT) of the signals was dominated by components around 150 kHz and very little activity was captured over 400 kHz. In order to reduce the size of the datasets and minimise the computing time, it was decided to use a sampling rate of 1 MHz and set the analogue antialiasing filter at 500 kHz. The high and low pass filter parameters in the acquisition system were set to 100 kHz and 500 kHz, respectively, with the preamplifier set to 40 dB for all acquisitions. From each experimental repetition and acquisition, 200,000 data points were extracted and used for the data analysis stage.

The sensor was supported with the PCI-2 Physical Acoustics data acquisition system manufactured by Mistras Group [2]. To ensure the repeatability of the measurements acquired over long periods, the sensor was attached to a fixed location on the plate. The testing procedure was iterated over several days to ensure the repeatability of the measurements.

The AE signal, which was recorded by the sensor and used to establish the particle size, comes from stress waves produced by the particles impinging on the target plate. Typically, these waves have relatively low amplitudes and high-frequency characteristics [5]. The dispensation source of the particles allows for a continuous stream for a set duration depending on the dispensed mass. This causes simultaneous impacts on the target plate, which in turn produce an AE signal consisting of overlapping AE burst events that can be characterised based on their amplitudes [2].

#### 2.2.2. Sample Preparation

**Figure 2:**
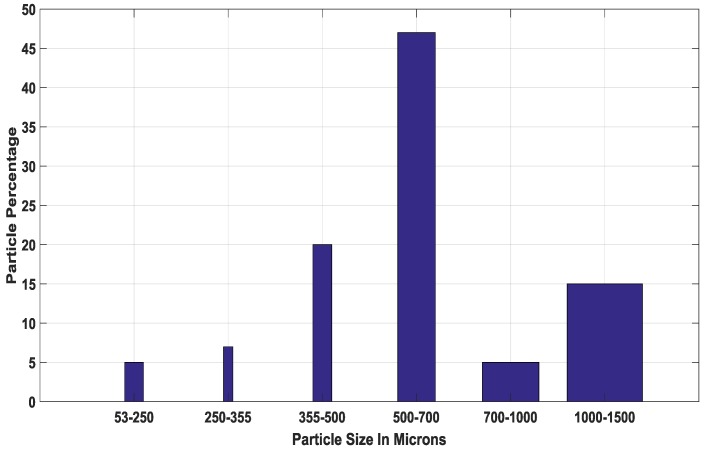
Ariel Actilift washing powder distribution obtained by sieving by weight.

**Figure 3:**
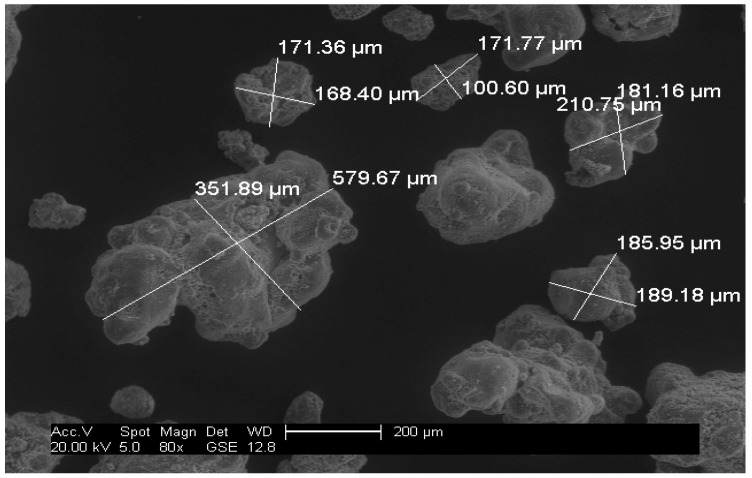
Micrograph of fine particles (53–500 microns).

**Figure 4:**
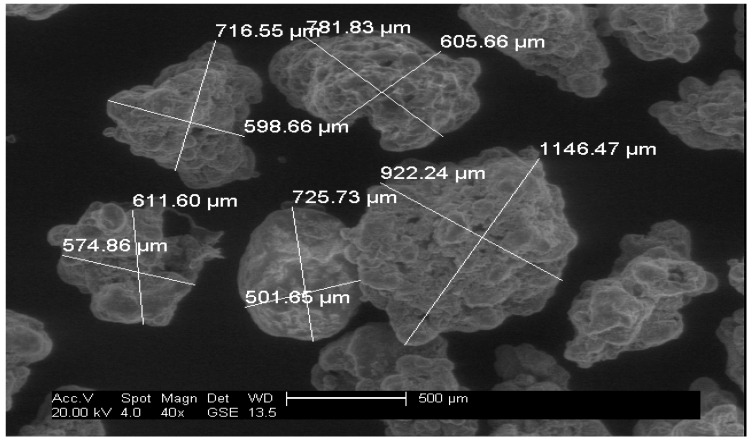
Micrograph of oversize particles (500–1500 microns).

Off-the-shelf Ariel Actilift washing powder was used for the experiments in this report and in the first instance was separated into groups of 53–500 microns (fine) and 500–1500 microns (oversize) particles by means of sieving. A distribution of the particles from a sample of the washing powder mixture can be seen in Figure 2; the bin divisions are a function of a standard sieve size and a micrograph of the two separated particle groups can be seen in Figure 3 and Figure 4.

From Figure 3 and Figure 4, it can be observed that the washing powder compound consisted of a mixture of regular and irregular sized particles. The samples were mixed in different ratios, as can be seen in Table 1, with a given mass of each powder ratio dispensed through the experimental rig five times to produce five experimental repetitions per powder mix ratio. These acquired AE data points were used to support the training of the signal processing method explained in Section 3.2.

## 3. Theoretical Model and Signal Processing Approach

### 3.1. Particle Sizing with the AE Theoretical Model

When a particle of a certain size impinges on a surface, elastic waves are produced that can be linked to the resulting forces that the impinging particle produces on the surface [10]. For a linear time-invariant (LTI) setup involving a plate serving as a wave propagation medium and a transducer to record the surface displacements produced by the stress waves, a mathematical expression was established by Buttle et al. [9] and can be seen in Equation (1) [9]:

(1) V(t)=S(t)∗G(t)∗R(t)

where S(*t*) = source function/source of the acoustic signal; G(*t*) = wave propagation function; R(*t*) = instrument response function; V(*t*) = AE voltage signal; “∗” represents a convolution.

In an LTI setup, the wave propagation medium can be said to have defined wave transmission properties, while transducers used to record AE events have been seen to have a flat response whose actual function is an output of the impedance matching process between the transducer itself and the wave propagation medium [3,10]. Thus, once the setup and definition of both functions have been carried out under certain conditions, the pair can be considered to be constant over a given interval [3,9,10].

Under the assumption that a given particle makes a normal and fully elastic impact, the source function S(*t*) represents the force–time history of the event and can be estimated using Hertz’s theory of contact for solid bodies [3,9,10].

Mathematically, the force produced by a particle impinging a plate can be estimated using Equation (2):

(2) $$S\left(t\right)=(f_{max}{\left(\sin\left(\frac{\pi t}{tc}\right)\right)}^{\frac{3}{2}},\;0\;\mathrm{for}\;0<t<t_{c}$$

where the particle contact time = $t_{c}$ = $4.53\left(4\rho_{1}\pi {\left(\left(\frac{\delta_{1}+\delta_{2}}{3}\right)\right)}^{\frac{2}{5}}\right)r_{1}v_{0}^{-1/5};$ and the peak compression force = $f_{max}$ = $1.917\rho_{1}^{\frac{3}{5}}{\left(\delta_{1}+\delta_{2}\right)}^{\frac{2}{5}}r_{1}v_{0}^{6/5}$ $\delta_{1}=\left(1-\mu_{i}^{2}\right)/\left({\pi E}_{i}\right)$.

The symbols E and μ represent Young’s modulus and Poisson’s ratio of the particle, respectively, while the subscripts 1 and 2 represent the particle and plate. $\rho_{1}$ refers to the mass density, $r_{1}$ denotes the equivalent radius and $v_{0}$ refers to the approach velocity of the particle. With sufficient knowledge of the mentioned variables, it is possible for the particle size to be theoretically estimated from the source function [10].

Hu et al. [10] observed in his studies that the maximum compression force produced by a particle hitting a plate can be said to be proportional to the peak compression force [10]. From Hu’s study, it can be said that the maximum absolute AE output signal can be linked to the peak compression force produced by the particle. Therefore, it can be hypothesised that a mean of these resulting forces produced by a particle impact above a carefully tuned and defined threshold can be correlated to a particle of a certain size [10].

Mathematically, this can be described as shown in Equation (3) for particle mixtures.

For case of particle mixtures:

(3) $$M=\frac{1}{n}\overset{n}{\underset{i=1}{\sum }}\left|x\right|$$

Threshold1<|*x*|>Threshold2.

where M = AE signal mean; n = number of values in the set being considered; x = AE voltage amplitude; i = indicates that the mathematical operation should start with the first number in the set being considered.

### 3.2. Signal Processing Method

To support the theoretical model proposed in Equation (3), an amplitude threshold approach will be used [11,12]. The threshold method involves the separation of the AE signal into component parts corresponding to the various particle groups present in the signal. Within each resulting signal part, a further threshold is added that searches the signal for an optimal region that contains information regarding particle size [11,12].

#### Threshold Tuning Stages

The following are the various stages involved in tuning the signal threshold using a two-particle mixture example.

**Figure 5:**
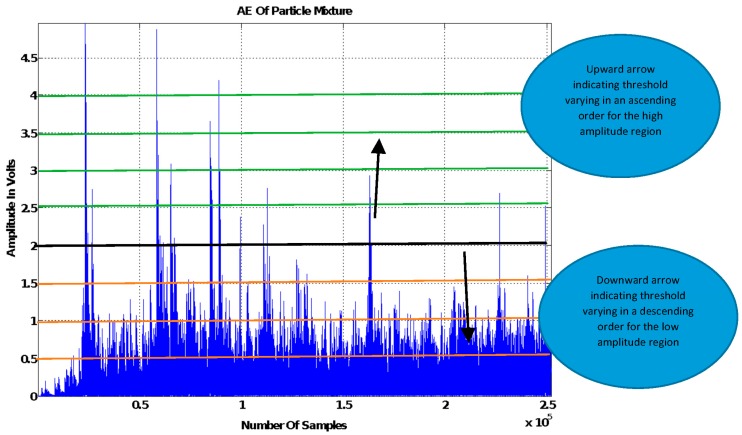
Visual illustration of the thresholding method for a mixture comprised of two constituent particle groups.

1. Expression of the signal in its absolute format |*x*| to rectify negative values.
2. Separation of the signal into high and low amplitude component parts corresponding to the impacts of the big and small particle distributions in the mixture, respectively.
  - For this to be achieved, a default threshold first needs to be implemented whose signal amplitude level is equal to the maximum produced from the resulting impact of the distribution of the small particles in the mixture.
  - In order to gain an accurate calibration of the default threshold, it is advisable to carry out a good number of experimental runs of unmixed material containing only the small particles. Figure 5 shows an example of a signal and a number of thresholds with the described default threshold represented by the black line.
3. Implementation of a varying threshold within each signal to scan across the length of each signal component part for a region containing signal information that can be linked to particle size.
  - The amplitude of the varying threshold should be varied in an ascending order in the high amplitude signal part and a descending order in the low amplitude signal part as can be seen in Figure 5. Due to the nature of the AE signals processed in this study, the threshold was adjusted by 0.5 V each time it was varied.
4. Extraction of the mean amplitude of the signal within the corresponding thresholds each time the amplitude of the varying threshold is changed, as shown in Equation (3).
5. Different mixture ratios containing a measured mass of both particles of interest should be formed, after which steps 3 and 4 should be repeated for each powder mix ratio. In this paper, the mix ratios shown in Table 1 were used. The acquired threshold amplitude mean should then be correlated to the respective powder mix ratio.
6. Each correlation plot produced from each threshold level should be validated using a new set of particle mixtures with the correlation plot that produces the highest estimation accuracy selected as the PSD estimation model. The amplitude parameters used to obtain the best correlation plot should be regarded as the optimal threshold level parameters.

## 4. Results and Discussion

Using the powder mix ratios shown in Table 1, the acquired AE signal from each repetition was analysed using a designed signal processing method where a threshold amplitude mean was obtained for each experimental repetition. This ensured the correlation plot comprised of a total of 45 experimental repetitions. The correlation plot with the best fit served as the PSD estimation model.

**Figure 6:**
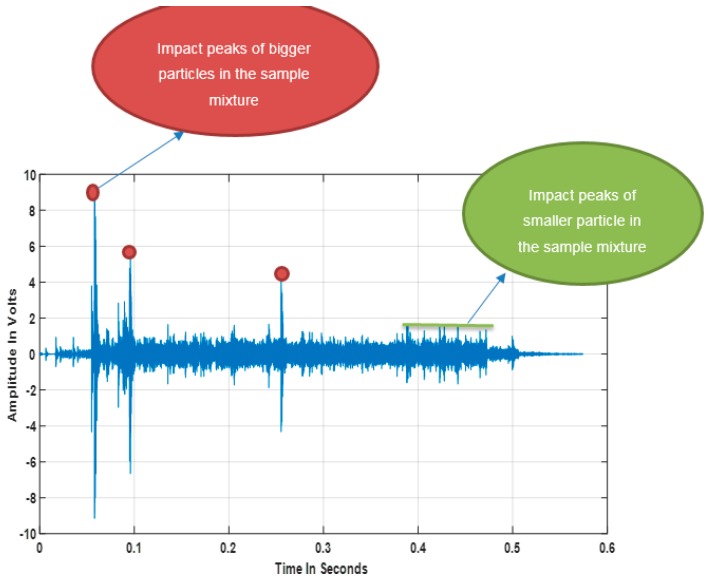
Sample the washing powder’s acoustic emission (AE) signal.

**Figure 7:**
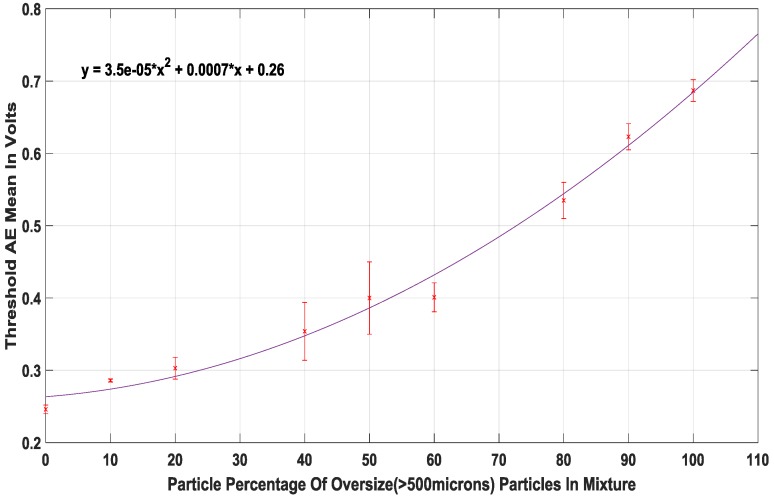
Correlation plot of oversize particles against AE amplitude mean with *R* = 0.97.

Using the described experimental and signal processing methods, the best correlation plot was seen to be located in the low amplitude signal section with a threshold of 1 V In the AE signal sample shown in Figure 6, it can be observed that the signal peaks have varying amplitudes, which is due to the broad particle distribution present in the washing powder compound as shown in Figure 3 and Figure 4. It can be noted from the annotation in Figure 6 that the significant impact peaks correspond to the bigger particles in the powder compound. The resulting correlation plot from the optimal threshold can be seen in Figure 7 and a table summarising the physical properties of the powders can be seen in Table 2.

The resulting data from the optimal correlation plot shows a quadratic trend between the particle mix ratio change and resulting threshold amplitude mean. The quadratic plot provides a good fit for the data and would allow for a better online computation time than the higher order polynomial fits that would provide an overfit of the data. In contrast to a previous study, a mixture of similarly sized particles yielded a linear correlation plot, suggesting that because the particles were physically similar, the change in AE was not steep as the particle mix ratio varied [9]. However, in this case, due to the different physical nature of the particle groups being considered, there are steeper changes in the trend displayed by the data points and as a result, a linear model provides a poor fit for the data. The quadratic curve that has been fitted to the data in Figure 7 provides an accurate way of modelling the steepness of the data induced by the nature of the particles. It is also worth noting that the correlation plot obtained in Figure 7 is unique to the nature of the powder that was used for its design.

With a new set of experimental mixtures, the PSD estimation model in Figure 7 was validated by comparing the estimation results given by the algorithm and the actual size percentage measured with the offline sieving method. An average absolute error * of 6% was obtained.

* Where the average absolute error is defined as: 

Estimation error = (actual particle %) − (estimated particle %)

Average absolute error = sum of estimation error/sum of no. of experiments

**Figure 8:**
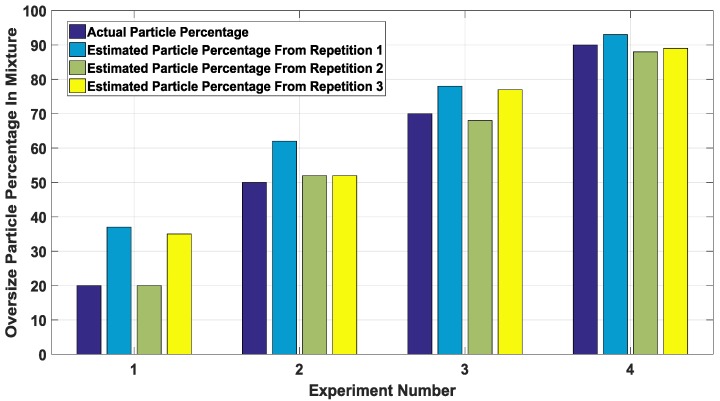
Particle size distribution (PSD) chart comparing the actual particle percentage to the amount estimated by the model.

Details of the set of validation experiments used can be seen in Table 3, with each validation mix ratio repeated three times. The chart in Figure 8 shows the results with the actual particle percentage for each experiment depicted by the dark blue bars, while the subsequent colour bars represent the repetitions carried out per mix ratio.

From Figure 8, it can be observed that the error decreases as the percentage of oversize particles increases in the mixture. This trend can be explained by the notion that bigger particles are easier to measure and thus make for a more accurate estimation by the PSD estimation model.

Even though the powder was classified into fine and oversize categories, each category comprised particles with various physical properties and geometries, which when mixed produced a distribution range of 1450 microns. This produced a rather complex signal with very high variability, as was recorded by the AE graph shown in Figure 6. This set of results suggests that under the tested conditions, the designed threshold approach to PSD estimation is capable of extracting particle size information from the AE signals of heterogeneous mixture compounds that are similar to the mixture considered in this study.

## 5. Conclusions

In this study, an investigation was made to determine the possibility of replicating the Procter and Gamble offline physical quality check procedure using AE sensing and a designed threshold-based signal processing method. The experiments were structured in a similar fashion to the partner company’s analysis, which is done by sieving and weighing a localised sample extracted from a washing powder batch to estimate the ratio of fine and oversize particles in the mixture. The results from the experiments carried out show that the mean AE amplitude from the optimal threshold correlates with the particle ratio change, and the validation exercises show that the correlation plot was able to estimate the PSD of the mixture with an average absolute error of 6%.

The results obtained from this set of experiments suggest that despite the variability in the acquired AE signal induced by the wide distribution of particles in the mixture, as well as their different physical properties, the designed threshold-based signal processing approach is capable of dealing with signals of this nature and ultimately estimating the related mix ratio. This provides evidence that AE can be used online for process monitoring of particle processes that produce powders similar to the set used for the experimental work in this study.

With the encouraging results obtained from the experiments in this study, subsequent work will involve the AE-based estimation of the full washing powder PSD, trying to replicate the results obtained by sieving in Figure 2.

## Figures and Tables

**Table 1:** Particle mix ratio percentage.

Mix Ratio	
Fines	Oversize
0	100
10	90
20	80
40	60
50	50
60	40
80	20
90	10
100	0

**Table 2:** Physical properties of the particles.

	Size Range (microns)	Bulk Density (g/cm^3^)	Percentage Bulk Density Difference
**Fines**	53–500	0.58	8%
**Oversize**	501–1500	0.63	

**Table 3:** Particle size distribution (PSD) estimation model validation: experiment details.

Experiment Number	Mix Ratio (Fines:Oversize)	Repetitions
1	80:20	3
2	50:50	3
3	30:70	3
4	10:90	3
